# Prevalence and Risk-Markers of Self-Harm in Autistic Children and Adults

**DOI:** 10.1007/s10803-019-04260-1

**Published:** 2019-10-26

**Authors:** Lucy Licence, Chris Oliver, Jo Moss, Caroline Richards

**Affiliations:** 1 School of Psychology, University of Birmingham, Edgbaston, Birmingham, B15 2TT UK; 2 School of Psychology, University of Surrey, Surrey, GU2 7XH UK

**Keywords:** Autism, Self-harm, Prevalence, Risk-marker, Affect, Impulsivity

## Abstract

Self-harm is purportedly common in autistic individuals, but under-researched, particularly in younger samples and those *without* intellectual disability. This study aimed to describe prevalence, profile and correlates of self-harm in autistic individuals without impairments in adaptive functioning. Parents of autistic participants (*n *= 83) completed questionnaires regarding the presence/topography of self-harm, demographic characteristics, autism severity, age of diagnosis, affect, activity levels and repetitive behaviour. 24.10% of participants engaged in self-harm. Self‐harm was associated with significantly higher levels of impulsivity, over-activity, negative affect, compulsive behaviour and insistence on sameness. Low mood and overactivity/impulsivity predicted the presence of self-harm, with the model correctly classifying 82.9% of cases. Findings highlight a role for impaired behavioural inhibition and low mood in the aetiological mechanisms underpinning self-harm in autism.

Autistic^1^ people are at heightened risk of developing psychiatric disorders compared to neurotypical individuals, with higher rates of depression and anxiety reported frequently (Lugnegård et al. 2011; Skokauskas and Gallagher 2010; Buck et al. 2014). Recent studies have highlighted self-harm (known as self-mutilation or non-suicidal self-injury) as a specific mental health concern in autism (Moseley et al. 2019; Maddox et al. 2017). Self-harm refers to the intentional act of physical damage to body tissue or self-poisoning, performed irrespective of the extent of suicidal intent or motive (NICE Clinical Guidelines 2011). Common forms of self-harm include: self-hitting, skin picking, self-biting self-cutting and self-burning (Pompili et al. 2015; Klonsky 2011). Prevalence estimates of self-harm in neurotypical samples vary between 5.5 and 17.2% (Swannell et al. 2014), with the presence of self-harm correlated with an elevated risk of suicide (Victor et al. 2015; Owens et al. 2002). Given the heightened rates of depression and suicidality in autistic samples (Zahid and Upthegrove 2017; Segers and Rawana 2014), self-harm in autistic individuals warrants further investigation.

Despite the clear rationale for research on self-harm in autism, studies of self-harm often explicitly exclude autistic participants (see Kim et al. 2015; Dickstein et al. 2015). One possible reason for this exclusion is an a priori definition of self-harm that categorises the typically repetitive self-injurious behaviours exhibited by autistic people as qualitatively different to self-harm shown by neurotypical individuals. Self-injurious behaviours (SIB) are defined as undesirable behaviours initiated by the individual that directly result in non-accidental personal physical harm (Murphy and Wilson 1985), including hitting oneself with an object or body part, head-banging, skin-picking, eye-pressing, scratching oneself and biting oneself (Richards et al. 2012, 2016; Murphy and Wilson 1985). These rhythmic and repetitive SIB are evaluated in studies of populations with *both* autism and intellectual disability (ID)/impaired adaptive functioning (Richards et al. 2012, 2016; Summers et al. 2017; Baghdadli et al. 2003; Weiss 2002). In these samples, SIB has predominantly been conceptualised within an operant learning framework (Summers et al. 2017) as a response acquired and maintained through reinforcement and mediated by sensory and environmental contingencies. Yates (2004) proposed these “stereotyped SIB” displayed by autistic/ID samples differ categorically from “impulsive” self-harm displayed by neurotypical samples; however this theory was derived from a developmental psychopathology framework rather than empirical data and as such warrants further investigation. Thus, to explore this hypothesised categorical difference it is necessary to describe self-harm in autistic individuals *without* ID, given the relative neglect of this area in current research. A more developed understanding of potential aetiological mechanisms underpinning self-harm in autism would assist in the assessment and treatment of this research-neglected but high-risk population.

In the first study comparing self-harm in autistic people *without* ID to a neurotypical sample, Maddox et al. (2017) reported that 50% of adult autistic participants had a history of self-harm. In an attempt to delineate self-harm from SIB, the authors also identified multiple similarities in self-harming phenomenology between the autistic and neurotypical adult samples, including adolescent age of onset, types and function of self-harm. More recently, Moseley et al. (2019) aimed to replicate Maddox et al’s descriptive results in a larger sample of autistic adults. Results regarding age of onset and forms of self-harm were analogous with both the neurotypical literature and Maddox et al’s (2017) results. However, neither study included children or adolescents, despite evidence of adolescence as a ‘risk’ period for self-harm in neurotypical and ASD/ID samples (Plener et al. 2015; Wilkinson 2013; Oliver et al. 1987). Given the high rates of self-harm and adolescent age of onset reported in Maddox et al. and Moseley et al., it is important to investigate self-harm in an autistic sample that includes participants below 18 years of age to gain a prospective understanding of the role of age in self-harm exhibited by autistic individuals *without* ID.

However, methodological challenges arise when translating studies of self-harm in autistic adults to self-harm in autistic children and adolescents. Approaches used to determine the absence of ID in previous survey studies, such as assessing employment status and completion of education, are unsuitable for use with child participants. ID diagnosis comprises both IQ scores below 70 and impairments in adaptive functioning (American Psychological Association 1994); as there are no current valid or reliable survey tools for measuring IQ, adaptive functioning is an appropriate proxy measure and is significantly predicted by IQ (Bölte and Poustka 2002; Kanne et al. 2011; Perry et al. 2009). Thus, to extend the literature on self-harm in autism to include child participants, the current study will assess adaptive functioning as a proxy measure of global ID. However, ID and adaptive functioning deficits do not always correspond, and indeed the association between adaptive functioning and IQ appears to become weaker as age increases (Pugliese et al. 2015). This may be the result of autistic adults without ID not acquiring chronologically age-expected adaptive skills as they age. Therefore the sample will be acknowledged as autistic individuals whose IQ is presumed in the normal range according to their adaptive function scores.

In addition to age, there are numerous other candidate risker markers and correlates of self-harm in autistic people which may develop our understanding of underlying mechanisms and inform future intervention. One putative correlate is age of autism diagnosis, as earlier identification of autism is critical for optimising lifetime outcomes. (Klin et al. 2015; Zwaigenbaum et al. 2013). Cassidy et al. (2014) reported a significant association between later age of autism diagnosis and elevated rates of suicidal ideation. Given the known association between self-harm and suicidal ideation in neurotypical samples, it is plausible that a later diagnosis of autism is associated with increased rates of self-harm, particularly if the same mechanisms underpin these behavioural (self-harm) and cognitive (suicidal ideation) symptoms. However, both Moseley et al. (2019) and Maddox et al. (2017) found no difference in self-harm prevalence dependent on age of autism diagnosis. Given this disparity, and the lack of samples including younger participants, there is an obvious need for further investigation into the role age of autism diagnosis may play in self-harm.

A second risk marker, autism severity, may also contribute to the increased likelihood of an individual exhibiting self-harm. Indeed, within autistic samples with ID both increased severity and quantity of autistic characteristics are associated with the presence of SIB (Rattaz et al. 2015; Matson and Rivet 2008; Baghdadli et al. 2003). However, Moseley et al. (2019) found no differences between current, historic and non-self-harmers in the number of autism traits measured using The Autism Spectrum Quotient (Baron-Cohen et al. 2001). Study of this association requires replication and extension to younger samples.

Furthermore, research into neurotypical samples identifies associations between self-harm and various demographic factors, which may in turn act as risk markers in autistic populations. Bresin and Schoenleber’s (2015) meta-analysis of 116 articles identified that females are significantly more likely to present a history of self-harm compared to males. Maddox et al.’s (2017) results suggest a similar trend, however, Moseley et al. (2019) found no evidence of a gender difference between participants who did and did not engage in self-harm. These equivocal findings associating gender differences with the presence of self-harm in autism require further investigation.

Finally, in addition to demographic differences associated with self-harm, behavioural characteristics may also act as risk markers or correlates of self-harm. In neurotypical samples, higher levels of impulsivity are associated with self-harm (Garisch and Wilson 2015). Research has shown that people with diagnoses of pervasive developmental disorders have significantly increased rates of impulsive behaviour and overactivity (Aman et al. 2008). Whilst the association between impulsivity and self-harm in autistic people without ID has not been explored, it is a known predictor of SIB in autistic samples including individuals with ID (Richman et al. 2013). Richards et al. (2012) found elevated levels of impulsivity and overactivity were associated with increased likelihood of SIB in a sample of autistic individuals with low adaptive functioning and that these traits predicated persistence of SIB over three years (Richards et al. 2016).

A second behavioural characteristic associated with SIB in autistic people with ID is repetitive and/or restricted patterns of behaviour (RRBs; Rojahn et al. 2016; Richards et al. 2016). SIB is sometimes conceptualised as a form of repetitive motor behaviour which may have been ‘shaped’ from stereotypic motor behaviours such as a hand-flapping (Lewis and Bodfish 1998 ; Tate and Baroff 1966; Rojahn et al. 2016). This correlation between repetitive behaviours and self-harm requires further investigation in autistic individuals without ID, given the proposed delineation between SIB as a repetitive behaviour associated with ID and self-harm as a behaviour associated with poor mental health in neurotypical populations. Relevant to this distinction, within neurotypical populations, low mood predicts the presence of self-harm (Garisch and Wilson 2015; Stallard et al. 2013). Moseley et al. (2019) found that depression predicted historic self-harm and that the most common function of self-harm was the regulation of low energy affective states. However, Maddox et al. (2017) reported no evidence of an association between depression and emotion regulation with self-harm in autistic adults. Thus, despite the growing evidence indicating associations between behavioural constructs and self-harm, the data are mixed, often contradictory, and frequently exclude autistic participants without adaptive impairments.

Therefore, there is a clear need to describe the phenomenology of self-harm in autistic samples of wider age ranges, including an examination of putative correlates including demographic and behavioural variables (repetitive behaviours, impulsivity, and mood). These data will enhance our understanding of self-harm in autistic people without adaptive impairments, directing future research and intervention strategies for this at-risk but research-neglected population.

*The aims of the present study were to*:

i. Describe the prevalence, forms and severity of self-harm in a sample of autistic children and adults without adaptive impairments. In line with previous studies in autistic adults, it is predicted rate of self-harm in autistic individuals will be higher than those reported in neurotypical samples.
ii. Investigate the association between demographic variables, age of autism diagnosis and autism severity with self-harm. It is predicted that self-harm will be related to increased autism severity and later age of diagnosis. Additionally, it is predicted that self-harm will peak during adolescence and likely be more common among female participants compared to males.
iii. Examine the association between potential behavioural risk markers and self-harm, including repetitive behaviours, impulsivity and overactivity, and affect. It is predicted that the presence of self-harm will be associated with higher levels of repetitive behaviour, impulsivity and overactivity, and more negative affect, and that these variables will also predict self-harm in autistic people without adaptive impairments.

## Method

### Recruitment

Autistic participants were recruited via the National Autistic Society (NAS); the leading UK charity for autistic people and their families. 288 parents/guardians of autistic individuals completed the questionnaire pack (19.63% return rate). A proportion of this dataset, participants whose adaptive function suggests lower than average IQ, was reported previously in Richards et al. (2012). Additionally, only 23% (n = 19) of the sample were adults and therefore given the ethical constraints of asking children about self-harm (Nairn and Clarke 2012), and the potential lack of capacity for younger individuals to complete the questionnaire pack independently, informant report methods, were deemed most appropriate.

### Procedure

All parents/guardians were provided with a cover letter, information sheet, consent form, demographic questionnaire and questionnaire pack. Measures were counterbalanced to minimise order effects. To prevent priming, the research was described as investigating behaviours associated with autism. Completed consent forms, demographic questionnaires, and questionnaire packs were returned by parents/guardians in a pre-paid envelope. Ethical approval to conduct this study was obtained from the Coventry NHS Ethics Committee.

### Measures

The questionnaire pack comprised the following informant-based measures. All measures were appropriate for use with children and adults with additional developmental needs (i.e. autism) and/or ID.

#### Demographic Questionnaire

A demographic questionnaire was included to obtain information regarding date of birth, gender, verbal ability (> 30 signs/words in their vocabulary), mobility and autism diagnosis.

#### The Wessex (Kushlick et al. 1973)

The Wessex was used as a proxy measure of ability, and is comprised of five subscales: self-help, mobility, continence, speech and literacy. The Wessex currently has no published validity data, but evidences modest inter-rater reliability at subscale level for both children and adults (Mean kappa value .62 and .54 respectively; Kushlick et al. 1973; Palmer and Jenkins 1982). It has also been argued that the Wessex is an appropriate and effective tool for large-scale questionnaire designs such as the present study (Oliver et al. 2012; Palmer and Jenkins 1982).

#### The Mood, Interest and Pleasure Questionnaire-Short Form (MIPQ-S; Ross and Oliver 2003)

The MIPQ-S was administered to assess affect. It is formed of two subscales, mood and interest and pleasure, and comprised of 12 items. The MIPQ-S has good internal consistency at both the full (Cronbach’s alpha coefficients: total = .88, mood = .79, interest and pleasure = .87) and subscale (alpha coefficient range for subscales .84 to .94) levels. The measure also has good test–retest (.97) and inter-rater reliability (.85). Concurrent validity between the MIPQ-S and the Aberrant Behaviour Checklist (ABC; Aman and Singh 1986) ranges between medium to strong (.36–.73; p < .001).

#### The Activity Questionnaire (TAQ; Burbidge et al. 2010)

The TAQ was used to assess participant activity levels, including behaviours indicative of impulsivity and overactivity. The measure is comprised of 18 items across three subscales: overactivity, impulsivity and impulsive speech. The TAQ currently has no published validity data, however demonstrates item level inter-rater reliability to range between .31 and .75 (mean = .56) and test–retest reliability to range between .60 and .90 (mean = .75). Both test–retest and inter-rate reliability indices for the three subscales and total TAQ score exceed .70. The subscales also evidence good internal consistency (alpha coefficients range for subscales .67 to .94).

#### The Repetitive Behaviour Questionnaire (RBQ: Moss et al. 2009)

The RBQ was used to assess repetitive behaviour across 19 items forming five subscales; Insistence on sameness, impulsive behaviour, stereotyped behaviour, repetitive speech and restricted preferences. Examination of the measures psychometric properties (Moss et al. 2009) indicates good test–retest reliability (coefficients range between .61 and .93), inter-rater reliability (coefficients range between .46 and .80) and internal consistency (alpha coefficients range for subscales .50 to .78). The RBQ also has good content and concurrent validity with the repetitive behaviour subscale of the Autism Screening Questionnaire (ASQ; Berument et al. 1999a, b).

#### The Social Communication Questionnaire-Lifetime Version (SCQ; Berument et al. 1999a, b)

The SCQ was included to assess autism behaviours displayed by participants. The measure is based on the Autism Diagnostic Interview (ADI-R; Lord et al. 1994), asking questions regarding development history to screen for ASD in children and adults. The SCQ is comprised of 40 items grouped to calculate a total score and three subscale scores; social interaction, communication and repetitive and stereotyped patterns of behaviour. Each item is scored to imply to presence (score = 1) or absence (score = 2) of behaviours or impairments associated with autism. The SCQ has two important scoring cut-offs, with a score of 15 or above suggesting the likely presence of autism and a higher score of 22 or above used to differentiate between individuals with autism and those with other pervasive developmental disorders. The measure demonstrates good concurrent validity with both the Autism Diagnostic Observation Schedule (ADOS: Lord et al. 2000) and the ADI-R (Howlin and Karpf 2004). Internal consistency is also good (α = .90 for the total scale). Rutter et al. (2003) provides evidence that 33 out of 39 items are able to differentiate between those with and without a diagnosis of autism, indicating good item level validity. To avoid confounds within the self-harm data, item 17 (Has she/he ever injured her/himself deliberately, such as biting her/his arm or banging her/his head?) was excluded from analysis when calculating participants’ total scores. All participants entered into the analysis, scored above the autism cut-off (15) *without* item 17.

#### The Challenging Behaviour Questionnaire (CBQ; Hyman et al. 2002)

The CBQ was directly developed from the Challenging Behaviour Interview (CBI; Oliver et al. 2003) and is used to examine a range of challenging behaviours present in the preceding month; self-injury, physical aggression, verbal aggression, destruction of property and stereotyped behaviour. Through the sub-items of the first question regarding the presence of self-injury, the measure also examines eight types of self-injurious behaviour, derived from Bodfish et al. (1995). These sub-items investigate the following forms: hits self with body part, hits self against surface or object, hits self with object, bites self, pulls, rubs or scratches self, inserts finger or objects. The measure also provides the option of ‘other’, presenting the opportunity to specify. Items evaluating self-injury were used to assess the presence and types of self-harm for the current study. Previous examination of the psychometric properties of the CBQ has evidenced good inter-rater reliability (coefficients ranging between .61 and .89; Hyman et al. 2002). Oliver and colleagues also demonstrated medium to strong (.19 to .68, p < .05) concurrent validity between the CBI and the ABC (Aman and Singh 1986).

### Participants

On return, questionnaire packs were screened and exclusion criteria applied. Participants were excluded if: (1) they were below the age of 4 years, as some measures were not appropriate for younger participants, (2) they did not have a confirmed diagnosis of autism from a relevant professional (Clinical Psychologist, Psychiatrist, Clinical Geneticist, General Practitioner, Paediatrician or Educational Psychologist), (3) 25% or more of items across measures were incomplete, (4) the participant had incomplete total scores on the Social Communication Questionnaire (SCQ; Berument et al. 1999a, b) or (5) they scored below the ASD cut-off on the SCQ. The Wessex was employed as a proxy measure of ability, and was therefore used to identify participants whose adaptive function suggests an IQ within the normal range. Participants who scored below nine on the self-help subscale of the Wessex (Kushlick et al. 1973) were assumed to have ID based on their adaptive functioning, and removed from the present analysis. Out of the 288 completed questionnaire packs, 149 participants were excluded as a result of scoring below nine on the Wessex, with 56 excluded for the other reasons listed.

After applying exclusion criteria, 83 participants were included in the study. Item 1 on the Challenging Behaviour Questionnaire (CBQ; Hyman et al. 2002; “Has the person shown self-injurious behaviour in the last month?”) was used to allocate participants to either the self-harm or no self-harm groups. Participants were aged between 4 and 45 years (Mean = 14.13, SD = 6.20). 30 participants were 11 years of age or below (36.1%: children), 34 were between 12 and 18 years of age (41%; adolescents) and 19 participants were over the age of 18 years (41%; adults). 67 participants were male (80.7%). 81 participants (97.60%) were fully mobile and 82 (98.80%) were verbal (> 30 signs/words in their vocabulary).

### Data Analysis

Kolmogorov–Smirnov tests were used to test for normality. Where data violated parametric assumptions (p < .05), non-parametric techniques were employed. To control for multiple comparisons, conservative alpha levels were used throughout (p < .01).

To investigate differences in demographic characteristics between those who did and did not engage in self-harm, categorical data were analysed using Chi squared statistics and ordinal data were analysed using Mann–Whitney U tests. Similar analytic techniques were utilised when investigating differences in autism behaviour and age of diagnosis. Fisher’s exact T was calculated if one or more cells had an expected count less than five.

Mann–Whitney U tests were employed to identify group differences between autistic individuals who did and did not engage in self-harm. Where data violated parametric assumptions effect size, *r*, was used as an alternative to standard difference statistics and then interpreted with Cohen’s *d* (Rosenthal et al. 1994; Fritz et al. 2012). A *d* value of + 1 is used to indicate that every datum point in a series is greater than every other datum point in the other series. A *d* value of − 1 is used to indicate that every datum point in a series is less than every other datum point in the other series. For determining the strength of effect size *r*, as interpreted by Cohen’s *d*, arbitrary cut-offs were assigned as followed based on Cohen’s recommendations: .1 = small, .3 = medium, .5 = large.

Finally, a forced entry binomial logistic regression was performed to examine the impact of variables that differed between groups on the likelihood of participants showing self-harm. Based on recommendations proposed in simulation studies of regression sample size (Peduzzi et al. 1996), to maximise power and increase parsimony, composite scores were created for subscales that measured the same global construct. Therefore, a repetitive behaviour composite score was created (the sum of compulsive behaviour and insistence on sameness scores), and the TAQ total score was used as an overactivity/impulsivity composite. These summed sub-scores were entered into the binomial logistic regression.

## Results

Of the 83 participants, 20 (24.1%) had engaged in self-harm in the preceding month. Table 1 presents the percentage of participants exhibiting each type of self-harm listed on the CBQ. Hitting self with body was the most common form reported, with over half (60%) of participants who engaged in self-harm showing this behaviour. A large proportion of participants (65.0%) engaged in more than one form of self-harm (Table 1). Table 1 also presents the frequencies of self-harm in those reported to display the behaviour. The majority of participants (55.6%) exhibited self-harm at least weekly.

**Table 1:** The types of self-harm, total number of topographies displayed by each participant, and frequency of self-harm displayed in the preceding month by autistic participants

	Percentage (N)
Topography	
Hits self with body	60.0 (12)
Hits self against object	25.0 (5)
Hits self with object	5.0 (1)
Bites self	50.0 (10)
Pulls self	35.0 (7)
Rubs/scratches self	50.0 (10)
Inserts	5.0 (1)
Other^a^	12.5 (2)
Number of topographies	
1	35.0 (7)
2	25.0 (5)
3	20.0 (4)
4	5.0 (1)
5	15.0 (3)
Frequency^b^	
Hourly	5.6 (1)
Daily	22.2 (4)
Weekly	55.6 (10)
Monthly	16.7 (4)

^a^‘Other’ total N = 16 participants due to missing data

^b^N = 19 for this analysis due to missing data

To investigate the second hypothesis, autistic participants who engaged in self-harm were compared to autistic participants who did not engage in self-harm on demographic variables, age of autism diagnosis and autism severity. Table 2 presents these results. Analyses revealed no significant differences between the self-harm and no-self-harm groups on any demographic variables. However, when examining the difference between the percentage of participants scoring above the higher cut-off of 22 on the SCQ (measure of autism severity), visual inspection of the data suggested the percentage was higher for those engaging in self-harm compared to those who did not (90.00% vs. 65.08% respectively), and indeed results approached significance (X^2^ = 4.59, p = .03).

**Table 2:** Demographic characteristics, autism severity and age of diagnosis compared between autistic participants who do and do not engage in self-harm

	Self-harm	No self-harm	U/χ^2^	*p* value
N	20	63		
Age (years)				
Mean	13.60	14.30	627.50	.98
(*SD*)	(4.36)	(6.70)		
Range	7–23	4–45		
Age (Categories)				
% ≤ 11	25	39.68	3.95	.14
% 12–18	60	34.92		
% > 18	15	25.40		
Gender				
% male	75	82.5	N/A^a^	.52
Mobility				
% mobile	100	96.83	N/A^a^	1.00
Speech				
% verbal	95^b^	100	N/A^a^	.24
SCQ total scores				
Mean	25.86	24.33	507.00	.19
(SD)	(4.80)	(4.88)		
Range	15–33.4	15–34		
Score 22 above on SCQ				
% above 22	90	65.08	4.59	.03
Age of diagnosis (months)				
Mean	102.11^b^	86.02	480.00	.19
(SD)	(63.49)	(71.66)		
Range	24–240	24–444		

Chi Square and Mann–Whitney U tests were employed to detect significant differences (p < .01). All analyses were two-tailed

^a^Fishers exact t calculated as 1 or more cells had expected count < 5

^b^N = 19 as missing data for Self-harm group

Furthermore, whilst analysis at both the continuous and categorical level of age yielded non-significant results, visual inspection of data suggested a trend towards higher levels of self-harm in the 12–18 year old age category (60%), compared to the ≤ 11 (25%) and ≥ 19 categories (15%).

In summary, there were no significant differences on measures of demographic variables, age of autism diagnosis and autism severity. However, categorical differences regarding a higher SCQ cut-off and adolescent age showed a trend towards significance.

To address the final aim of the study, Table 3 reports the differences in behavioural characteristics between participants with and without self-harm, including measures of mood, repetitive behaviour and activity level. Those presenting self-harm evidenced significantly higher total scores on the RBQ and TAQ, and significantly lower total scores on the MIPQ-S, with participants who engaged in self-harm reported to have significantly lower mood compared to those who did not. These individuals with self-harm also scored significantly higher on subscales measuring compulsive behaviour and insistence on sameness. Finally, individuals reported as engaging in self-harm scored significantly higher on all three sub-scales (overactivity, impulsivity and impulsive speech) of the TAQ compared to those reported to not be engaging in self-harm. All significant differences were associated with medium effect sizes (Cohen’s *d* range: .34–.41).

**Table 3:** Comparison of measures of affect, repetitive behaviour and activity/impulsivity between participants who did and did not engage in self-harm

Measure Subscale	Median scores for participants		U Score	*P* value	Effect size *r* interpreted through Cohen’s *d*
	Self-harm N = 20	No self-harm N = 63			
MIPQ-S					
Mood	**17.00**	**20.00**	**278.00**	**< .001**	**−** **.41**
Interest and pleasure	11.500	14.00	428.500	.032	**−** .24
MIPQ total score	**29.00**	**20.00**	**344.500**	**.002**	**−** **.33**
RBQ					
Stereotyped behaviour	5.00	3.00	453.500	.106	**−** .18
Compulsive behaviour	**8.00**	**3.00**	**300.500**	**.001**	**−** **.36**
Insistence on sameness	**4.00**	**3.00**	**322.000**	**.002**	**−** **.34**
Restricted preferences	7.00	4.00	395.500	.050	**−** .22
Repetitive language	6.00	4.00	411.500	.074	**−** .2
RBQ total score	**32.00**	**18.00**	**313.500**	**.002**	**−** **.35**
TAQ					
Overactivity	**20.5650**	**10.00**	**275.500**	**< .001**	**−** **.41**
Impulsivity	**18.500**	**11.00**	**288.500**	**< .001**	**−** **.40**
Impulsive speech	**9.00**	**5.00**	**322.500**	**.002**	**−** **.34**
TAQ total score	**47.00**	**25.00**	**254.500**	**< .001**	**−** **.44**

Median scores and Mann–Whitney U statistics are reported. Significant differences (p < .01) are highlighted in bold. Where data violated parametric assumptions effect size *r* was used as an alternative to standard difference statistics and then interpreted with Cohen’s *d* (Rosenthal et al. 1994; Fritz et al. 2012). For determining the strength of effect size r as interpreted by Cohen’s *d*, arbitrary cut-offs were assigned as followed: .1 = small, .3 = medium, .5 = large

In summary, autistic individuals who engaged in self-harm had significantly different total scores on all behavioural measures (RBQ, TAQ and MIPQ-S) compared to those who were not reported to engage in self-harm. Specifically, these participants had significantly higher compulsive behaviour, insistence on sameness, overactivity and impulsivity scores, as well as significantly lower scores on the mood subscale.

Due to the dichotomous nature of the dependent variable (self-harm), a forced entry binomial logistic regression was performed to assess the impact of independent variables on the likelihood of participants engaging in self-harm. The model contained three independent variables that were associated with the presence of self-harm in the analyses above: mood subscale score, repetitive behaviour composite score and overactivity/impulsivity composite score. The full model containing all predictors was statistically significant (X^2^ = 27.70, df = 3, p < .01). A Hosmer and Lemeshow test also suggested the model was a good fit for the data (p > .05). These results indicate that the model was able to successfully distinguish between participants engaging in self-harm and those not engaging in self-harm. The model as a whole accounted for between 29.00% (Cox and Snell R) and 43.00% (Nagelkerke R) of the variance in self-harm, and correctly classified 82.9% of cases.

Table 4 displays the results of the logistic regression. The overactivity/impulsivity composite made the most statistically significant contribution to the model, with an odds ratio of 1.07. This suggests that the odds of autistic individuals with higher levels of impulsivity and overactivity were 1.07 times more likely to engage in self-harm when controlling for other factors in the model. Mood also significantly contributed towards the model, with an odds ratio of .79. This suggests that the odds of autistic individuals with lower mood were 1.26 times more likely to engage in in self-harm when controlling for other factors in the model.

**Table 4:** Binomial logistic regression predicting the likelihood of an autistic individual engaging in self-harm

	*B*	SE	Wald	*df*	*p*	Odds ratio	95.0% CI for odds ratio	
							Lower	Upper
Mood	**−** **.23**	**.10**	**5.26**	**1**	**.022**	**.79**	**.65**	**.97**
Repetitive behaviour	.04	.03	1.53	1	.216	1.04	.98	1.11
Overactivity/impulsivity	**.07**	**.02**	**8.52**	**1**	**.004**	**1.07**	**1.02**	**1.13**

Significant predictors (p < .05) are highlighted in bold

## Discussion

The prevalence, type and associated characteristics of self-harm in autistic people *without* adaptive impairments were examined in this study. Compared to previous research, this study was the first to recruit a sample including a wide age range of children, adolescents and adults, as well as to consider a variety of putative correlates. Findings have clear clinical utility, by providing the first evidence of affective and cognitive correlates of self-harm in autistic people without impairments in adaptive functioning.

The results of the study revealed a relatively high prevalence of 24.1% for self-harm. This is considerably higher than rates of 5–17% reported within neurotypical samples (Swannell et al. 2014) but lower than the 50% prevalence reported previously by Maddox et al. (2017) in autistic adults. Perhaps this is the result of the current study including younger children within the sample, as self-harm is reported to have an adolescent age of onset (Moseley et al. 2019; Maddox et al. 2017). Additionally, as a consequence of data being collected as part of a wider study including participants without the capacity to self-report (see Richards et al. 2012), and the inclusion of younger children in the present paper, information regarding participants’ self-harm was obtained via parent-report methods. Whilst often utilised in autism research due to ethical constraints, parent-participant report discrepancies are widely acknowledged in the neurotypical literature (De Los Reyes 2011, 2013; Achenbach 2006), in neurodevelopmental populations (Fisher et al. 2014), and particularly when investigating behavioural and emotional problems (Chen et al. 2017). Interestingly, various researchers have commented on the secrecy of self-harm, particularly in more severe cases, in which individuals often conceal evidence of the behaviour and do not disclose it to families or professionals (Chandler 2018; Best 2006; Crouch and Wright 2004). Therefore, the private nature of self-harm may have resulted in parents/guardians being unaware of the behaviour, leading to the phenomena being under-reported in the current study. Nonetheless, even with these considerations, the prevalence rate reported in the present study is significantly higher than that reported within neurotypical samples, and alludes to autistic individuals whose adaptive functioning score suggests an IQ within the normal range being at an elevated risk of developing self-harm. Additionally, despite skin-picking and self-biting being concealed forms of self-harm, which may be unnoticed by parents, 50% of informants reported that participants engaged in self-scratching and self-biting. This makes them the joint second most common topographies of self-harm displayed by the sample, which is a similar pattern to self-injury seen in samples with co-morbid ID (Richards et al. 2012), autistic samples without ID (Moseley et al. 2019; Maddox et al. 2017) and in neurotypical self-harm (Pompili et al. 2015; Klonsky 2011). Results therefore highlight the clinical need for services to address self-harm in autistic individuals.

The present study also described frequency of self-harm. Self-harm was most commonly reported to occur weekly, followed by daily, monthly and hourly. This follows patterns of self-harm presented in neurotypical samples (Ross and Heath 2002). Despite the similar frequency of self-harm in autistic and neurotypical samples reported here, autistic people report significantly more challenges accessing support for mental health difficulties (Crane et al. 2019; Camm-Crosbie et al. 2018), with a noted shortage of professionals trained to support autistic individuals presenting with self-harm and suicidality (Raja 2014). The results of this study further support the need for training and investment in the clinical workforce to meet this need.

The profile and type of self-harm behaviours displayed by autistic individuals without adaptive impairments was described in this study. Hitting oneself with their own body, scratching themselves, and biting themselves were the most commonly reported forms of self-harm. The majority of autistic people who showed self-harm, engaged in more than one form of self-harm. Results replicate patterns exhibited in autistic samples *with* impaired adaptive functioning that suggests an IQ below the normal range (Richards et al. 2012), and are similar to those reported in adult autistic samples Moseley et al. 2019; Maddox et al. 2017). However, despite self-cutting being a prominent behaviour reported within these existing autism studies, and also being recognised as the most common form of self-harm within the neurotypical population (Ross and Heath 2002), the current results do not reflect this trend. A potential caveat suggests the need for caution when interpreting these results, as data were derived from a larger investigation of self-harming behaviours in a wide sample with autism, resulting in self-harm being assessed using a measure which did not include an explicit option for self-cutting. However, inclusion of an ‘other’ option provided protection against subsequent threats to validity as it allowed the opportunity for parents/guardians to report additional forms of self-harm not listed, and indeed one participant was reported to engage in self-cutting upon visual inspection of these data. Future research should utilise a specific measure of self-harm (for example, The Deliberate Self-Harm Inventory; Gratz 2001) and consider complementing informant report tools with self-report measures to more fully describe the types of self-harm exhibited in this population.

Results of the present study also evidenced no significant association between age of autism diagnosis and the likelihood of an autistic individual engaging in self-harm. This supports previous findings (Moseley et al. 2019; Maddox et al. 2017) despite evidence that age of diagnosis does have an influence on suicide ideation (Cassidy et al. 2014). This alludes to potential differences in the aetiological mechanisms between these two frequently co-occurring mental health needs, suggesting the possible requirement for them to be considered, assessed and treated differently. This is interesting given that results of the present study also supported Moseley et al.’s (2019) findings associating self-harm with significantly lower mood. Low mood is associated with suicidality within the autistic population and individuals with Asperger Syndrome (Richa et al. 2014; Mayes et al. 2013; Mukaddes and Fateh 2010). Therefore, low mood might mediate self-harm and suicidality displayed by autistic individuals, whilst age of diagnosis only correlates with risk of suicide, further investigation is needed to understand the associations between these clinical features.

Potential further explanation for this interesting result regarding age of autism diagnosis may be derived from The Interpersonal Model of Suicide, which was proposed by Joiner (2005) and advanced by Van Orden et al. (2010). This model seeks to account for the observation that the large majority of people with suicidal thoughts do not subsequently attempt suicide. Within the parameters of this model, self-harm likely escalates the risk of suicide for an autistic individual by increasing ‘acquired capability’ to perform the act; however, this alone is likely not enough to create suicidal risk. Additionally, thwarted belongingness and perceived burdensomeness are core constructs within the model, and are predicted to induce hopelessness which may then lead to suicidal desire. The addition of acquired capability to this may consequently result in a suicide attempt (Chu et al. 2017). Perhaps age of autism diagnosis affects one of these contributing constructs for suicide rather than the development of self-harm behavior, and as such warrants further investigation.

Differences in autism severity between those with and without self-harm trended towards significance, with results suggesting that individuals scoring above 22 on the SCQ were more likely to engage in self-harm. Whilst this putative correlate might allude to clinically relevant mechanisms, results are weak and not supported by previous research (Moseley et al. 2019). Given the relationship between elevated levels of autistic behaviour and SIB in people with ID (Richards et al. 2012), possible explanations might draw upon functional differences between self-harm and SIB, with communication difficulties commonly implicated in the ID literature (Chiang 2008; McClintock et al. 2003). Both heightened ability and reduced autism symptom severity are associated with superior language acquisition and skills (Marrus and Hall 2017 Wodka et al. 2013; Ray-Subramanian and Ellis Weismer 2012), and thus it is possible that ability may mediate the relationship between autism severity and self-harm. Indeed, this might also explain why Moseley et al. (2019) did not find any relationship between autism severity and self-harm, as this study used a measure of autistic traits which is primarily suitable for individuals with good verbal ability. However, support for this model requires direct investigation of these characteristics. Additionally, caution must be taken in the interpretation of these results as the sample was derived from parent support groups, and as such autism severity data may have been biased by the inclusion of families who were receiving higher levels of advice and support compared to the general population. However, as a large proportion of the results are comparable to those reported by Maddox et al. (2017) and Moseley et al. (2019), findings using this recruitment strategy remain valid.

The results of the present study revealed no significant gender or age differences in the likelihood of engaging in self-harm. Visual inspection of results suggested a trend towards higher levels of self-harm within adolescence (12–18 years), which is reflective of the neurotypical population (Plener et al. 2015; Fliege et al. 2009). However, it is interesting that this result does not emerge as prominently as the adolescent peak seen in the neurotypical population. Results therefore suggest the need for further investigation into the role age has in the development of self-harm among autistic individuals, to investigate whether adolescence is an at-risk age or not. The lack of gender difference in the present study between those with and without self-harm supports recent findings by Moseley et al. (2019) and data drawn from samples with autism and impaired adaptive functioning that suggests an IQ below the normal range (Richards et al. 2012, 2016; McClintock et al. 2003). However, these results are inconsistent with findings presented by Maddox et al. (2017) and the neurotypical self-harm literature (Bresin and Schoenleber 2015) which report a higher prevalence of self-harm among females. These discrepancies may be the consequence of the gender imbalance within autism research. A preponderance of autistic males is clearly evidenced with recent estimates purporting a ratio of around 3:1 autistic males to females (Loomes et al. 2017), and as such research populations are often skewed accordingly. However, emerging evidence suggests that autism is underdiagnosed in females, particularly in those without ID (Bargiela et al. 2016; Lai et al. 2015; Rivet and Matson 2011). Indeed, the dataset analysed within the current study was predominantly male (80.72%). Therefore, both conflicting results and an evident gender bias create challenges for delineating the relationship between gender and self-harm, indicating the need for future research utilising a more representative and perhaps stratified sampling strategy.

Interestingly, our results indicate a strong relationship between self-harm and a feature of the core autism phenotype; repetitive behaviours and restricted interests. Analysis revealed overall RBQ scores were significantly higher in the self-harm group, and highlighted in particular elevated levels of compulsive behaviours and insistence on sameness, which parallels correlates of SIB in the ID literature (Wolff et al. 2013; Oliver et al. 2012; Lewis and Bodfish 1998). These findings may suggest that self-harm exhibited by the autistic population has repetitive and stereotypic features. Additionally, RRBs have a clinically relevant association with anxiety in autistic people. Recent research has proposed a link between RRBs with anxiety, in particular insistence on sameness and compulsions (Gotham et al. 2013; Kamp-Becker et al. 2009). The role of anxiety has been implicated in the aetiology of self-harm in neurotypical individuals (Hawton et al. 2002). Importantly, Moseley et al. (2019) found anxiety to be a potential risk-marker of self-harm in autistic adults. Therefore, heightened levels of RRBs associated with self-harm might be indicative of anxiety. Identification of causal associations between anxiety and self-harm could assist early intervention programmes by highlighting anxiety as a therapeutic target to reduce self-harm. An additional contributing factor may be intolerance to uncertainty; a dispositional characteristic that stems from negative beliefs regarding uncertainty and the implications resulting from uncertainty (Dugas and Robichaud 2007). Indeed, intolerance to uncertainty has been found to be closely related to both autistic symptomology and RRBs (Vasa et al. 2018), and to anxiety in autism (Boulter et al. 2014; Neil et al. 2016; Rodgers et al. 2016a, b). Intolerance to uncertainty has also be associated with depression, which is the most commonly co-occurring mental health need with self-harm (Carleton et al. 2012). Therefore, future research into anxiety and self-harm among autistic people should investigate the contribution of intolerance to uncertainty as a potential variable that may increase the risk of developing self-harm.

Finally, analyses revealed key differences in behavioural characteristics between autistic individuals who did and did not engage in self-harm. Individuals reported to engage in self-harm evidenced elevated levels of overactivity, impulsivity and impulsive speech. These results support previous findings associating behavioural characteristics of overactivity and impulsivity with self-harm in neurotypical samples (Garisch and Wilson 2015; Stallard et al. 2013) and with SIB in autistic samples with ID (Richards et al. 2012, 2016; Aman et al. 2008). Despite limited power, multivariate analysis revealed both low mood and elevated levels of impulsivity/overactivity were able to significantly predict allocation to either the self-harm or no self-harm groups, correctly classifying 82.9% of cases. Previous research acknowledges the relationship between impulsivity and inhibition (Logan et al. 1997), and as such increased levels of impulsivity and overactivity might be behavioural indicators of impaired inhibition. Therefore, current results lend tentative evidence to the roles of negative affect and impairments in executive function in aetiological models of self-harm within autism. Investigation involving direct experiments of executive functioning and affect dysregulation are now required, as confirmation of their role within self-harm would have substantive clinical utility for the assessment and early intervention of self-harm.

The current study advances the existing autism literature by being the first to describe the prevalence and profile of self-harm in children and adults within this population, as well as identifying potential risk-markers for the development of self-harm. Whilst various important demographic and behavioural variables were investigated, the study did not investigate other known associated factors, such as sleep problems (Hysing et al. 2015), substance abuse (Nitkowski and Petermann 2011), exercise (Klonsky and Glenn 2008), diet (Ayton et al. 2003) and anxiety (Hawton et al. 2002), creating a potential caveat in the research. Future research should aim to include these potentially important variables in order to assess their contribution to the development of self-harm among autistic individuals.

In summary, results of the current study demonstrate a high prevalence of self-harm within autistic individuals whose IQ is presumed in the normal range according to their adaptive function scores, with a variety of associated behavioural characteristics. Findings present similarities to both neurotypical samples and samples with ID and therefore allude to the need to draw upon aetiological models derived from both populations, in order to further our understanding of the self-harm in this under-researched group. Due to the obvious clinical utility of results, future research is warranted to explore these associated behavioural characteristics further.

## Footnotes

1 Authors acknowledge the ongoing debate surrounding autism terminology. Whilst person-first language is often regarded as portraying respect towards individuals with developmental disabilities (Clarke et al. 2017), the neurodiversity movement champions descriptions portraying autism as a way of being, and rejects the idea that a persons’ autism can be separated from their identity (Singer 1999). Additionally, Kenny et al. (2016) recently reported the majority of autistic individuals and their families to endorse the term ‘autistic’. Therefore, the descriptors “autistic person/people/individual” and ‘autism’ are used in the current paper.
